# Arterial hypertension in the chronic evolution of migraine: bystander or risk factor? An overview

**DOI:** 10.1186/s10194-024-01720-7

**Published:** 2024-02-05

**Authors:** Federico Mazzacane, Gloria Vaghi, Matteo Cotta Ramusino, Giulia Perini, Alfredo Costa

**Affiliations:** 1 Department of Brain and Behavioral Sciences, University of Pavia, 27100 Pavia, Italy; 2 Unit of Behavioral Neurology, IRCCS Mondino Foundation, Via Mondino 2, 27100 Pavia, Italy; 3 Headache Science & Neurorehabilitation Center, IRCCS Mondino Foundation, 27100 Pavia, Italy

**Keywords:** Migraine, Arterial hypertension, Endothelial dysfunction, Chronic migraine, Chronicization

## Abstract

**Background:**

Several risk factors are associated with the chronic evolution of migraine. Clinical and preclinical studies have provided data about the role of hypertension (HT) as one of the potential modifiable risk factors of chronic migraine (CM). This review is focused on the biological and clinical evidence supporting common mechanisms underlying HT and migraine and the potential role of HT in the transition from episodic to chronic migraine.

**Methods:**

We conducted a narrative review from a literature search covering the available evidence from studies investigating: i) the role of HT in the transition to CM in clinical practice; ii) the biological mechanisms potentially underpinning the association between HT and evolution to CM; iii) the role of antihypertensive medications in migraine prophylaxis.

**Results:**

HT proved to be at the base of multiple mechanisms underlying migraine and migraine chronicization. Endothelial dysfunction, blood–brain barrier alterations, calcitonin gene-related peptide signaling, and renin–angiotensin–aldosterone system dysregulation are involved in the worsening effect of HT on migraine frequency, and the role of HT in the transition to CM is supported by clinical observations.

**Conclusions:**

The observed evidence supports HT contribution to CM evolution due to shared pathophysiologic mechanisms. While a bidirectional influence appears to be ascertained, data are still lacking about the one-way role of HT as direct risk factor for CM transition. Further research is needed to confirm a causal role of HT in this process.

## Background

Migraine is a chronic disease with an estimated 1-year prevalence of 15% worldwide [1]. It is one of the most important causes of disability, being the first cause in women below the age of 50 [2], and it carries relevant socio-economic and daily life burdens [3].

Migraine is classified as episodic or chronic based on headache frequency over the previous three months. Episodic migraine (EM) refers to individuals who experience less than 15 headache days per month, while chronic migraine (CM) is characterized by 15 or more monthly headache days, with at least 8 days per month with migraine features (ICHD-3, 2018) [4]. CM can be complicated by medication overuse headache (MOH), classified on the basis of the intake of analgesics, triptans, opioids, ergotamine or their combination [4, 5].The threshold for monthly days of use is set at 15 days/month in the previous three months for simple analgesics, while it is lowered to 10 days for the other classes [4]. CM is the most disabling phenotype across the migraine spectrum, as it severely impairs patients’ quality of life and represents a major determinant of the direct and indirect costs of the disease [6]. The disease burden is even increased when considering resistant and refractory migraine forms [7]. Migraine-related disability should be considered preventable, as effective acute and preventive treatments are now available [8].

CM often develops in patients previously suffering from EM. Numerous studies suggest a transition model in which migraine progresses from EM to CM [9] with a 1-year rate progression of about 2.2–3.1% [10, 11]. Progression is driven by both non-modifiable and modifiable factors, which should all be identified and addressed in migraine management. Risk factors encompass socioeconomic status, female gender, obesity, major life events, asthma, non-cephalic pain, head and neck injuries, snoring and insomnia, as well as suboptimal medical therapies [10–16] This data is further supported by recent systematic reviews and meta-analyses [17, 18] that highlight the role of depression [RR 1.58, 95%CI 1.35–1.85], headache frequency [≥ 5 days/month: RR 3.18 95%CI 2.65–3.82; ≥ 10 days/month: RR 5.95, 95%CI 4.75–7.46] and medication overuse [RR 8.82, 95%CI 2.88–27.0] [17].

Another risk factor that has recently gained further importance is arterial hypertension (HT), one of the cornerstones of the well-known association between migraine and vascular diseases. Since HT is a treatable condition, the recognition of its role in migraine progression may have an important clinical significance, leading to a proper management and consequent decrease of migraine progression rate, as well as of associated disability and costs. This narrative review aims at a summary and critical reappraisal of clinical and preclinical evidence available so far in order to evaluate the role of HT in CM transition.

## Hypertension and the biological mechanisms of migraine chronicization

CM is a complex disorder affecting 1–2% of the global population [3], usually manifesting as a progressive headache worsening with transition from low-frequency to high-frequency attacks[19]. Though still not completely understood, accepted underlying mechanisms contributing to CM development include dysfunction of descending pain modulation areas (especially the periaqueductal gray -PAG); hypersensitivity of trigeminal system leading to central sensitization, expressed by a reduced nociceptive threshold; increased cortical excitability; blood–brain barrier (BBB) alterations; and chronic neurogenic inflammation [19, 20].

HT seems to play a critical role in this context, as its consequences on the cardiovascular homeostasis could negatively influence migraine course and lead to CM [21–23]. Indeed, previous studies supported the role of vascular diseases in migraine pathophysiology, even though their specific interconnections are still unclear. Regarding a possible common genetic background, only single-nucleotide polymorphisms linked with vascular function have been found in migraine patients [24]. From a pathophysiological point of view, migraine and vascular diseases, especially HT, share some underlying mechanisms, such as autonomic dysregulation [25], deranged renin-angiotensin system (RAAS) [26] and endothelial dysfunction (ED) [27]. They also share comorbidities within the metabolic syndrome spectrum, namely elevated body mass index (BMI) [28], insulin resistance [29], and dyslipidemia [30]; all conditions associated to an increased cardiovascular risk.

In the following sections we will review the main biological mechanisms potentially underlying the association between HT and migraine, with a particular focus to CM evolution, and the clinical evidence of the association between HT and transition to CM.

### Endothelial dysfunction

The endothelium is one of the first and main target of HT-induced damage. Under homeostatic conditions it has antithrombotic, anti-inflammatory and antioxidant functions. It is also involved in vessel tone and blood pressure control via production, and balanced interplay, of vasodilatory and vasoconstrictive substances, such as nitric oxide (NO), endothelin 1 (ET-1) and prostacyclin, and inactivation of other factors, such as serotonin and bradykinin [31, 32]. When the endothelium is negatively affected by detrimental factors like HT and reduced vasodilator bioavailability, a proinflammatory and procoagulant condition known as ED may develop [33]. ED can alter the release of endothelial mediators, including growth factors, cytokines, adenosine triphosphate (ATP), and NO, partly responsible for the sensitization of trigeminal neurons [34]. HT is also directly related to ED through increased vascular stiffness, reactive oxygen species (ROS) production and consequent inflammation [21, 35].

Beyond a passive involvement of cerebral vasculature during migraine attacks, previous literature have increasingly recognized endothelium as a main actor in migraine pathophysiology. This is supported by the evidence of vasodilatory reduction and contextual increase of vasoconstrictive substances during the attacks (namely ET-1, metalloproteinase 9, soluble intercellular adhesion molecules) [36], as well as lower levels of endothelium self-repairing progenitor cells (EPCs) and higher ET-1 levels in migraine subjects compared to healthy controls [37].

Nonetheless, the causal and temporal relationship between ED and migraine is far to be determined. It is currently still unclear whether ED may be a consequence of repeated migraine attacks or one of its causes, and whether it only acts as a migraine trigger or also as a key factor for its chronic evolution. In addition, it remains to be elucidated whether ED may be one of the factors driving the association between stroke and migraine.

The endothelium is a potential target of several noxious factors in migraine patients, particularly those suffering from migraine with aura.

Oxidative stress represents a major cause of tissue damage, when it exceeds the endothelium antioxidant capacity it can lead to ED. An environment characterized by increased ROS production determines maladaptive vascular changes, namely increased platelet aggregation and loss of vasodilation, increased inflammation and smooth muscle cell growth [38]. ROS directly inhibit NO activity and activate the PI3K/ras/Akt/MAPK pathway, resulting in inhibition of endothelial nitric oxide synthase (eNOS) mRNA expression and eNOS activity [38–40]. Previous evidence showed increased oxidative stress in migraine sufferers represented by higher levels of oxidized LDL (oxLDL) and malondialdehyde (end product of lipid peroxidation), and decreased activity of various antioxidant enzymes [41].

In addition, migraine is associated with an inflammatory state, as indicated by high levels of cytokines, like interleukin (IL) 1β, IL-6, and Tumor Necrosis Factor -α, as well as Endothelial Cell Specific Molecule 1 [42], but this state might be either the cause or the result of oxidative stress.The strong link between ED and migraine is further supported by genome-wide association studies. Indeed, the vast majority of genomic loci associated with migraine are also linked to vascular function [34].

From this perspective, the occurrence of HT-mediated disruption of physiological endothelial and vascular homeostasis may have a detrimental effect on migraine, enhancing the underlying disease mechanisms. HT may act as a continuous endothelial noxious stimulus favoring further ED, and thereby creating a predisposed environment for migraine progression from episodic to chronic [21, 22, 43]. Nonetheless, the hypothesis that ED could be determined by a common driver, namely metabolic dysfunction, acting in both CM and HT, cannot be ruled out and may represent a potential confounder.

### Insulin resistance and metabolic dysfunction

Insulin resistance (IR) is a condition of a diminished physiological response to normal insulin levels, that requires increased insulin production to maintain sufficient intracellular glucose concentrations. IR is a factor of the metabolic syndrome along with hypertension, dyslipidemia, abdominal obesity and systemic inflammation [44].

IR has been associated to migraine in several studies in the last two decades [45, 46] and it is also a recognized cause of ED [47]. In fact, IR leads to a selective impairment of insulin-mediated NO production, through a PI3K-mediated pathway. Simultaneously, insulin-mediated ET-1 production is even enhanced through a MAPK-dependent signaling [48].

This hypothesis was strengthened by a recent case–control study on 30 newly diagnosed migraine patients that revealed a significantly higher mean values of serum insulin and Homeostatic Model Assessment (HOMA) index in migraineurs compared to a control group (*p* = 0.049 and *p* = 0.01, respectively). Moreover, migraine patients had a higher frequency of insulin resistance (46.7% vs 16.7%, *p* = 0.012) and metabolic syndrome (43.3% vs 16.7%, *p* = 0.024) [49].

Focusing on CM, a cross-sectional study conducted by Fava et al. found a higher prevalence of IR in women with CM compared to EM (24% vs 9.4%, *p* = 0.03). After adjusting for potential confounders, IR remained independently associated with CM (aOR 3.1, 95%CI 2.7–3.7, *p* = 0.001), with an event stronger association in patients with concomitant obesity (aOR = 12.4; 95%CI 11.0–14.6, *p* = 0.001). HT (aOR 1. 4 95%CI 1.1–1.7, *p* = 0.05) was another independent predictor of CM [46].

### Blood–brain barrier dysfunction

Another interconnection between migraine chronicization and HT is represented by a blood–brain barrier (BBB) disruption, i.e. a condition linked to both HT and CM. The detrimental effect of HT on BBB has been observed in both animal models and clinical studies [50–52]. ED, immune cells and metalloproteinases (MMPs) activities are responsible for the disruption of BBB integrity with consequent direct cellular damage and invasion of the central nervous system (CNS) by immunity cells [50, 52, 53]. In fact, MMP-9, MMP-2, MMP-3, and MMP-1 serum levels are increased in both animal models of HT and in hypertensive patients, with MMP-9 being one of the most involved agent in BBB disruption [54]. Cortical spreading depression (CSD), a hallmark feature of migraine, is also known to alter the permeability of BBB, at least partially, through the activation of MMPs. An increased frequency of migraine attacks could thus determine a progressive disruption of the BBB homeostasis that may be further enhanced by the concomitant presence of HT, with its known detrimental effect on BBB integrity. On the other hand, HT induced BBB dysfunction may facilitate the triggering pathogenetic mechanism that starts the migraine attack [18]. Despite the reported association among HT, migraine and BBB dysfunction, their causal relationships is still unclear. In fact, the potential role of BBB dysfunction in triggering migraine has been proposed in previous studies, but even the more recent neuroimaging techniques failed to definitively prove it [55].

### Calcitonin gene-related peptide (CGRP)

The role of HT in migraine chronicization may also be ascribed to HT effect on the calcitonin gene-related peptide (CGRP), a key mediator in migraine pathophysiology. This 37-amino acid neuropeptide has recently become even more important with the development of effective treatments targeting its pathway [56]. CGRP is centrally involved in activation and sensitization of afferent trigeminal nociceptors of the trigeminovascular system, the central element of the head-pain processing pathway. Previous in vivo human and animal studies demonstrated CGRP presence in areas strictly related with migraine pathophysiology and pain transmission both in the CNS (namely in hypothalamus, thalamus, and cerebellum) and the peripheral nervous system (namely sensory neurons in the trigeminal ganglion and unmyelinated C fibers and small myelinated Aδ fibers, as well as dorsal root ganglia) [57, 58].

CGRP is known to contribute to neurogenic inflammation, release of neuron sensitizing agents and dural vasodilation [56]. Sustained CGRP release is involved in the transition to CM via the induction of peripheral sensitization [34, 59]. Moreover, CM patients were found to show higher CGRP levels in peripheral blood (74.90 ± 28.29 pg/mL) compared to EM patients (46.37 ± 15.21 pg/mL) and healthy controls (33.74 ± 16.10 pg/ mL), making them a potential marker of CM (CM vs EM: *p* = 0.001; CM vs HC: *p* < 0.001) [20].

Beside its well-known role in migraine, CGRP exerts cardiovascular beneficial effects thanks to its vasodilatory and cardioprotective properties [60]. Notably, previous studies showed elevated CGRP plasma levels in patients with essential HT, and HT due to phaeochromocytoma and primary aldosteronism [61]. This was interpreted as a possible compensatory reaction aimed at reducing blood pressure elevation [61]. Thus, the compensatory CGRP chronic increased release in HT may be a triggering factor of sensitization and inflammation, two events underlying migraine and possibly favoring its chronic evolution. This provides another potential link between HT and CM, though specific studies directly investigating this hypothesis are still lacking [60]. In addition, previous investigations showed conflicting results on CGRP elevation in HT [62], probably due to the fading away of CGRP compensatory response as disease progresses.

### Renin-angiotensin-aldosteron system (RAAS)

The RAAS is another intriguing element in the complex interplay between HT and CM. RAAS activity in the CNS modulates sensory and pain information, emotional and behavioral responses, stress, anxiety, learning and memory [63]. RAAS dysregulation is crucial in HT development, progression, and organ damage [64, 65]. It has also been associated to neurogenic inflammation, susceptibility to oxidative stress, ED, and neuromodulation of nociceptive transmission [26] all crucial processes in migraine pathophysiology as well. RAAS role in migraine mechanisms was also supported by the evidence of RAAS hyperactivation and higher angiotensin II levels in migraine patients [66]. Moreover, angiotensin receptors (AT1 and AT2) are expressed in the most important CNS loci involved in nociception and pain modulation, such as the anterior cingulated cortex, prefrontal cortex, thalamus, PAG, amygdala, nucleus accumbens and spinal cord [63]. The PAG represents an endogenous analgesic network able to control pain via the enkephalin releasing neurons projecting to the raphe nuclei, in the brainstem, and inhibiting the nociceptive afferents of the trigeminal nucleus caudalis. Recent evidence suggests that a disruption of PAG control on the trigeminovascular system may favour migraine attacks [67] and migraine chronicization [19, 63]. AT receptors located at these sites can be a target for the deranged RAAS activity seen in HT that can favor transition to CM through central sensitization.

The effect of RAAS on CNS can also be mediated by the overexpression of TNF-alfa induced by RAAS. Indeed, TNF-α has been found to promote both peripheral and central sensitization [68, 69]. Other potential contributing mechanisms are meningeal mast-cells activation [70]and oxidative stress [71]. The role of RAAS in migraine and CM is further supported by the efficacy of angiotensin receptor blockers (ARB) and ACE-inhibitors in migraine prophylaxis [72–76].

**Fig. 1:**
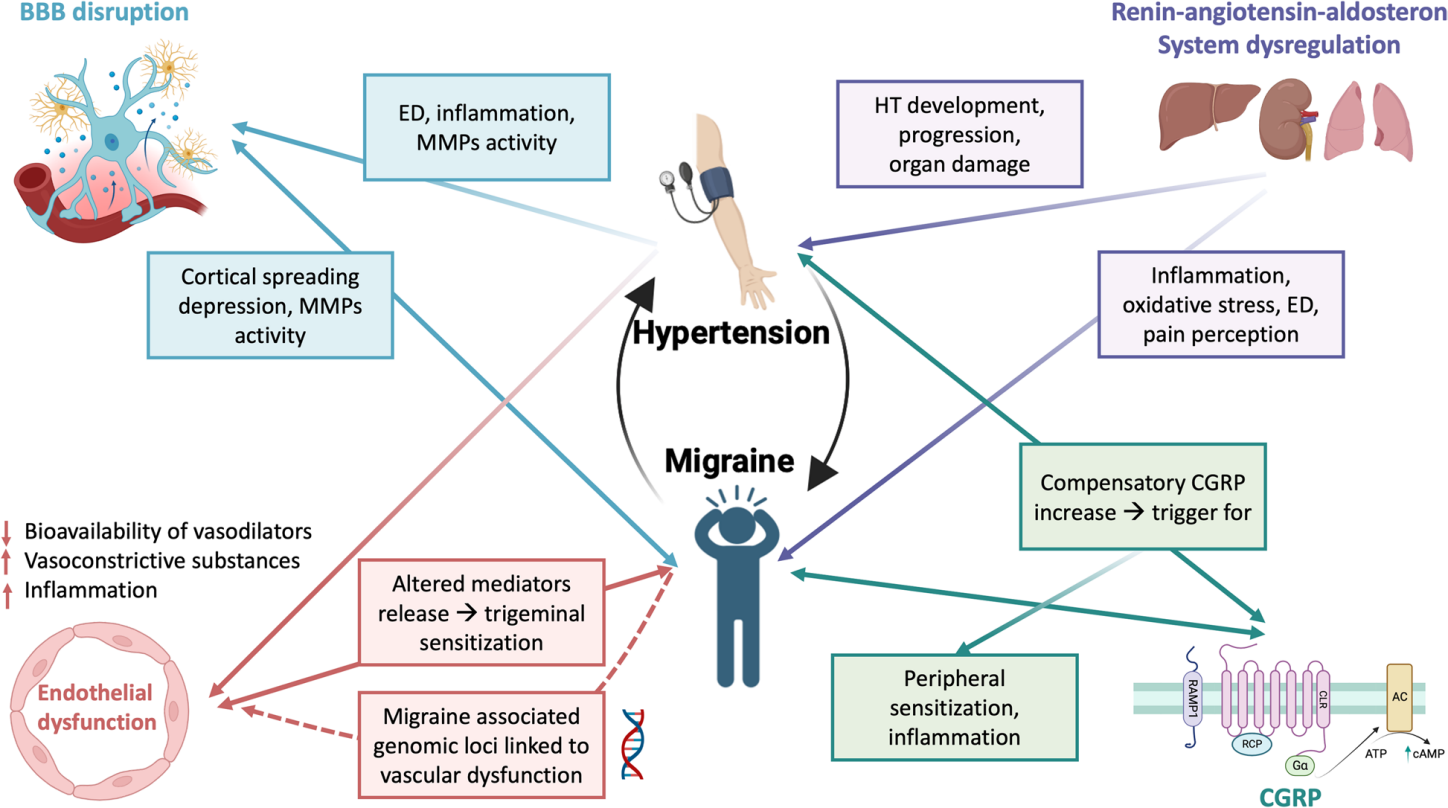
Possible pathophysiological mechanisms linking migraine and hypertension (HT). Endothelial dysfunction (ED) is a condition characterized by reduced vasodilator bioavailability, and a proinflammatory and procoagulant state. It may be a cause and a worsening factor of HT. ED is also associated with migraine but is still unclear whether it is a consequence or a cause of migraine attacks. Migraine-related genomic loci were found to be linked to vascular function. ED and MMPs activation could also lead to blood brain barrier disruption, with consequent neuroinflammation. These conditions are present in both HT and migraine patients, where MMPs activation could be determined by cortical spreading depression. RAAS dysregulation is associated with neurogenic inflammation, oxidative stress, ED and it is strictly related to HT development and progression. RAAS regulatory sites are expressed in areas involved in nociception and pain modulation, as the PAG, an endogenous analgesic network which is now considered as a possible migraine generator. CGRP is a key factor in migraine pathogenesis leading to peripheral sensitization. As a compensatory mechanism it was also found to be chronically elevated in HT patients, possibly triggering sensitization and inflammation in migraine. Abbreviations: BBB blood brain barrier, CGRP calcitonin gene related peptide, CSD: cortical spreading depression, ED endothelial dysfunction, HT hypertension, MMPs metalloproteinases, RAAS renin–angiotensin–aldosterone system. Created with biorender.com

All the mentioned mechanisms are summarized in Fig. 1.

## Hypertension and migraine chronicization: evidence from clinical studies

Associations between both EM, CM, vascular risk factors and cerebrovascular diseases have been extensively reported in clinical studies [30, 77, 78]. While the older investigations reported inconclusive results, the most recent observations appear to support a direct association between HT and increased migraine frequency [79, 80]. Nonetheless, available researches have several limitations, mainly linked to study design (cross-sectional or retrospective) that prevent from properly assessing a definite causal relationship between migraine and hypertension. In large population-based cohorts, as in the Northern Manhattan Study, a significant association between HT and migraine was reported [81]. These findings were confirmed in a very recent study showing a higher prevalence of HT among midlife women with a history of migraine [82]. Two other large prospective studies reported consistent results. The Women’s Health Study is one of the few with a prospective design. It involved over 20,000 middle-aged and older women and found that women presenting migraine history had an increase of 16% of the relative risk of presenting HT compared to those without migraine history [83].

Furthermore, evidence points to a direct association between HT and migraine frequency. The prevalence of HT appears to be higher in patients with CM than EM, as reported by Buse et al. (33.7 vs. 27.9%, OR = 1.23, 95% CI: 1.03–1.47) [79]. Moreover, data from the Women’s health study revealed that women with at least one migraine attack per week had a 30% increased risk of HT when compared to those with less than six migraine attacks per year [83]. These results were in line with another large 5-year prospective Finnish population study showing that migraine patients had a 1.4-fold increased risk of developing hypertension compared to people without a baseline migraine diagnosis [77]. However, no direct association between hypertension and migraine progression could be established as no evident association between baseline hypertension diagnosis and migraine development was found. An association between HT and migraine (OR 1.51, CI95% 1.4–1.6) was also found in the MAST study, a recent prospective web-based survey conducted on the US population including 15,133 subjects with migraine and 77,453 controls. Interestingly, among migraineurs, HT was directly associated with the numbers of headache days per month (15–20 days OR 1.52 (1.29, 1.8), > 21 days OR 1.37 (1.13, 1.66); reference 1–4 days) with higher prevalence of HT in patients with CM [80].

Older retrospective studies already suggested a role of HT in CM development, with the limitation of a different classification of headache phenotypes [22]. More recently, a large, randomized, case–control study conducted by Bigal et al. investigating somatic comorbidities associated with development of CM, pointed to a strong relationship between HT and CM (OR 6.9, CI 3.1–15.9, p < 0.0001). The association was also evident for HT and MOH (OR 2.9, CI 1.3–6.5, *p* = 0.01) [84]. This study strengthened previous evidence with the added value of including two control groups, namely EM and chronic post-traumatic headache patients. Interestingly, the authors found that patients with CM had multiple associations with somatic comorbidities, including HT, while MOH patients had very few of such associations [84]. While in MOH development medication overuse and psychological comorbidities may take the lion’s share [85], the above-mentioned positive association between HT and both CM and MOH conditions corroborate the role of HT in the process of chronicization and underlines the importance of investigating and eventually treat this comorbidity. All these results are further confirmed by multiple observational studies that showed a higher prevalence of HT in CM than in EM patients [79, 86, 87].

Some evidence pointing towards a higher susceptibility of developing CM in patients with HT was provided by a retrospective study by Manzoni et al. [88]. The authors analyzed 315 medical records of migraine patients with a mean follow-up of almost 15 years. Interestingly, women that subsequently evolved to CM showed a higher rate of arterial HT during the previous follow-up period (38.7% vs 17.9%, *p* < 0.01) [88]. No differences between patients who developed CM compared to those who did not were found in the prevalence of head injuries, dysthyroidism, colitis, allergy, insomnia, anxiety, and panic attack disorder. These findings, in line with a previous study by Bigal and collaborators [84], highlighted the importance of HT among somatic disorders that may contribute to chronicization of migraine [88]. Nevertheless, these results should be interpreted with caution, as the study from Manzoni was limited by the retrospective design, the women-restricted small sample size and the lack of control for potential confounders.

The studies focused on the relationship between migraine and hypertension are summarized in Table 1.

**Table 1:** Characteristics of clinical studies focused on the relationship between hypertension and migraine

Author, year	Study design	Aims	Sample size	Population	Identified risk factors (OR)	Limitations
Bigal, 2002 [84]	Randomized case–control design	To identify risk factors for CM evolution	791	CM – MOH CM EM cPTH	HT prevalence higher in CM compared to EM and cPTH CM vs EM OR 6.9 (95% CI 3.1—15.9) CM vs cPTH OR 5.1 (95% CI 2.7 – 11.1)	
Bigal, 2010 [78]	Cross-sectional	To profile CV risk in migraine and ascertain CV events in migraine vs controls	6 102 migraineurs 5 243 controls	MwA MA HC	Migraineurs have higher risk of developing HT OR 1.4 (95% CI 1.3–1.6)	• Self-reported data • No collection of concomitant CV risk factors • Cross-sectional
Buse, 2010 [79]	Cross-sectional	To assess differences in comorbidities between CM and EM	24 000	CM EM	HT prevalence higher in CM 1.23 (95% CI 1.03–1.47)	• Self-reported data • Cross-sectional
Gipponi, 2010 [87]	Cross-sectional	To identify risk factors for CM evolution	1 483	EM CM—MOH cTTH	HT prevalence higher in CM CM 16.2% vs EM 7.3% and vs cTTH 6.6%, *p* < 0.01	• Cross-sectional
Manzoni, 2012 [88]	Retrospective (10-year follow up)	To evaluate risk factor for CM evolution	315	MwA → MwA MwA → CM	HT prevalence higher in pts with evolution to CM 38.7% vs 17.9%, *p* < 0.01	• Retrospective • Mostly women • Small sample size
Entonen, 2014 [77]	Prospective (5-year follow-up)	To identify association between migraine and HT evolution	13 454	Random sample of Finnish population	Higher risk of developing HT in migraine pts OR 1.4 (95% CI 12–1.7)	• Self-reported data • No evaluation of concomitant CV risk factors
Fagernæs, 2015 [89]	Prospective (11-year follow up)	To evaluate association between migraine and HT	13 852	Random sample of the Nord-Trondelag County	Inverse correlation between HT and migraine development Per 10 mmHg increase in Systolic BP: OR 0.8 (95% CI 0.8–0.9); Diastolic BP: OR 0.98 (95% CI 0.8–1.2)	• Self-reported data • No data on medications • Selection bias (low % of pts who completed the final questionnaires)
Gardener, 2016 [81]	Cross-sectional	To investigate association between migraine and HT	1 338	Random sample of the Northern Manhattan community	HT higher prevalence in migraine OR 1.76 (95% CI 1.2—2.5)	• Self-reported data • Cross-sectional design
Rist, 2018 [83]	To evaluate association between migraine and incident HT	To evaluate risk of incident HT in migraine	29 040 (all women)	MA MwA HC	Higher risk of developing HT in women with migraine MA RR 1.09 (95% CI 1.0 -1.2) MwA RR 1.21 (95% CI 1.1 -1.3)	• Self-reported data • Observation bias • women only
Buse, 2020 [80]	Cross-sectional	Better understanding of migraine comorbidities	92 586	Migraine HC	Higher prevalence of HT in migraine 15–20 days OR 1.52 (1.3, 1.8), > 21 days OR 1.37 (1.1, 1.7); reference 1–4 days	• Self-reported data • Cross-sectional • Selection bias
Cotta Ramusino, 2022 [90]	Cross-sectional	To investigate HT contribution to CM evolution	48	CM EM HC	Altered brain vessel wall reactivity in CM and HT pts. Greater decrease in cerebral blood flow velocity in EM pts with associated HT *p* = 0.037	• Cross-sectional • Ongoing treatments
Faubion, 2023 [82]	Cross-sectional	To assess association between migraine and hypertension	5 708 (all women)	Random sample on data registry	Higher prevalence of HT in migraine aOR 1.31 (95% CI 1.1 – 1.6)	• Self-reported data • Cross-sectional • women only

*Abbreviations:* *BP* blood pressure, *CI* confidence interval, *CM* chronic migraine, *cPTH* chronic post-traumatic headache, *cTTH* chronic tension type headache, *HC* healthy controls, *HT* hypertension, *MwA* migraine without aura, *MA* migraine with aura, *OR* odds ratio, *pts* patients, *RR* relative risk

An interesting insight in the role of HT in worsening headache frequency came from a large meta-analysis of randomized placebo-controlled trials of four different classes of blood pressure-lowering drugs (thiazides, β-blockers, ACE inhibitors and angiotensin II receptor antagonists) in which data on headache were also reported [91]. Indeed, all four classes, sharing the same blood pressure (BP) lowering effect despite the different mechanisms of action, were associated with a reduced incidence of headache (thiazides: OR 0.71, 95%CI 0.56–0.89; beta-blockers: OR 0.47 95%CI 0.35–0.63; ACE-inhibitors: OR 0.74 95%CI 0.62–0.88; angiotensin II receptor blockers: OR 0.65 95%CI 0.56–0.75). The greater reduction seen in beta-blocker trials appears to be consistent with their efficacy in migraine prevention, also involving a BP-independent effect [92]. In addition, a statistically significant dose–response correlation between headache frequency and diastolic BP lowering was seen for all the four classes of medication, with a diminished headache prevalence in trials achieving a greater diastolic BP reduction [91]. Nonetheless, it should be noted that no specific definition of headache type is provided in the study and migraine frequency assessment was not the primary outcome of the cited studies.

Taking into account all the limitations, the evidence that all antihypertensive agents reduced headache frequency, including classes without a known role as migraine preventive therapies (namely thiazides), may led us speculate about the existence of a therapeutic effect potentially related to the BP-lowering action alone. This hypothesis is further strengthened by a very recent systematic review and meta-analysis on the effect of different classes of antihypertensive medications, including alpha-blockers, ARB, ACE inhibitors, beta-blockers, and calcium channel blockers in migraine prevention. All investigated classes of anti-hypertensive medications proved to be significantly effective, with multiple effective treatment in the same class, including clonidine, candesartan, atenolol, bisoprolol, propranolol, timolol, nicardipine, and verapamil [93]. This study supports the idea that hypertension control in migraine patients with a comorbid elevated blood pressure may represent an additional option for migraine treatment. Adequately designed clinical trials are needed to confirm these results.

**Fig. 2:**
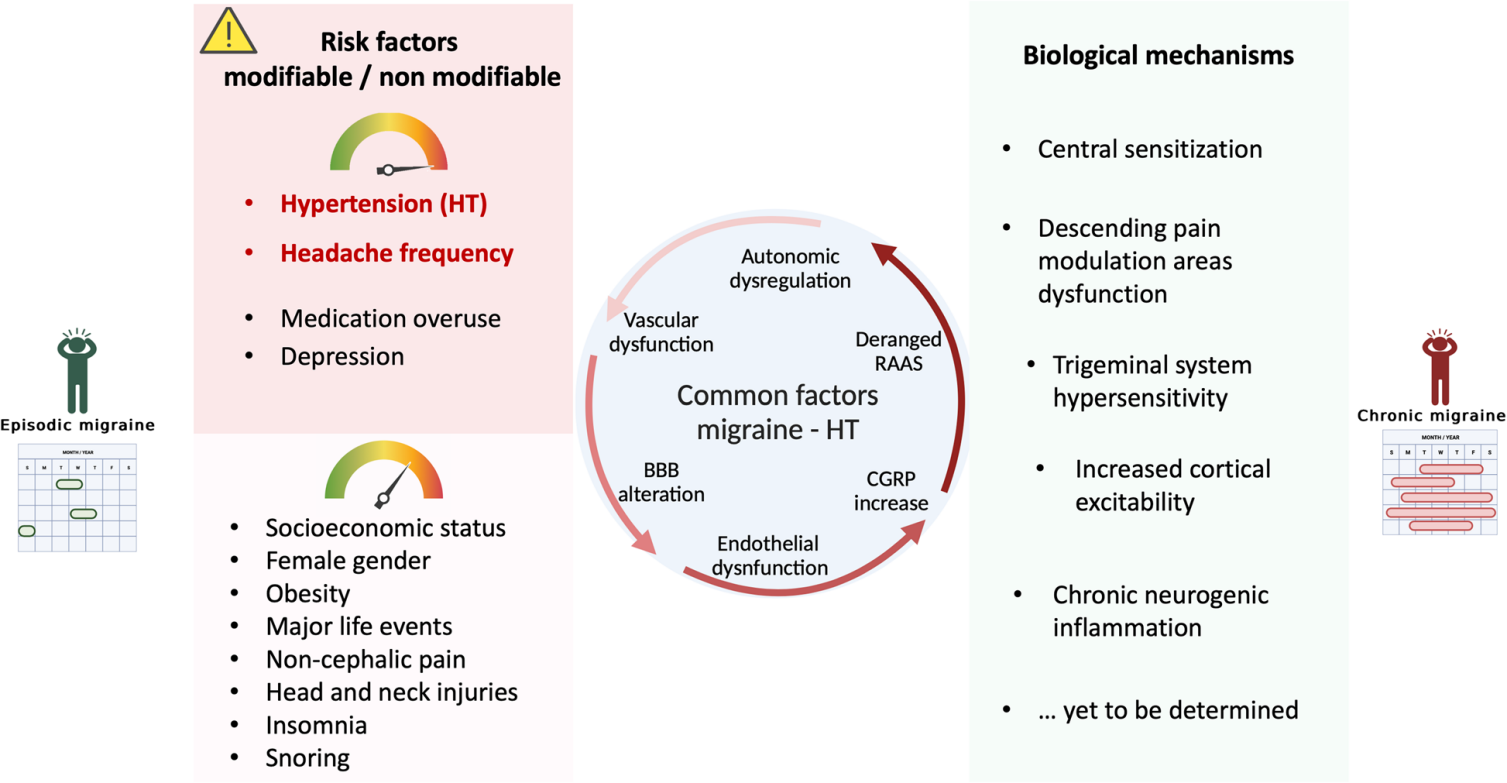
Migraine modifiable and non/modifiable risk factors and biological mechanisms associated with transition to chronic migraine (CM). Common pathophysiological factors between migraine and hypertension are represented in the middle. Abbreviations: BBB blood brain barrier, CGRP calcitonin gene related peptide, CSD: cortical spreading depression, ED endothelial dysfunction, HT hypertension, MMPs metalloproteinases, PAG periaqueductal grey, RAAS renin–angiotensin–aldosterone system. Created with biorender.com

Even though the potential causative link between HT, especially uncontrolled HT, and migraine chronicization is still not clearly defined, further clues also came from the recent literature. The existence of an underlying vascular dysfunction in patient with HT and CM was addressed in a recent clinical study by our group [90], whereby patients with EM and CM were further divided between hypertensive and non-hypertensive subjects. Cerebral vascular reactivity was assessed by transcranial doppler ultrasound measuring cerebral blood velocity (CBV) in the middle cerebral artery, before and after glyceryl trinitrate (GTN) administration. Patients were also investigated with a 24-h BP monitoring. We found that CBV decline was significantly more pronounced in patients with CM and HT. There was also a trend toward a diminished physiological nocturnal BP dipping in CM patients [90]. These findings point to a shared cerebrovascular dysregulation with an altered cerebral vessel wall reactivity in both CM patients and HT patients. Though no causal relationship could be established from this data, HT-related vascular damage seems to be synergic to migraine-induced vascular inflammation. By acting as an additive detrimental factor on the cerebral vessels of migraine patients, vascular insults may lead to an increased attack frequency and subsequent chronicization [31, 94]. Nonetheless, these results should not be taken as conclusive, as they do not rule out the possibility of a bidirectional interaction between HT and CM. Some studies also suggested an association between CM and risk factors potentially contributing, and further leading to, HT, namely reduced physical activity, depression and obesity [95, 96]. The chronic pain associated with CM may be another contributor, as its association with hypertension was proved by previous studies [97]. According to this view, transition to CM may worsen BP control through its associated comorbidities and lead to ED, which through a vicious cycle may eventually result in a further increase in migraine frequency (Fig. 2).

On the other hand, some studies even pointed to an inverse relationship between HT and migraine, as shown by an analysis of the data from Nord-Trondelag Health Survey 1995–1997 (HUNT 2) and 2006–2008 (HUNT 3) [89]. These findings, however, have not been confirmed in any further study.

We can thus conclude that the relationship between HT and CM evolution has been a relevant topic over the last decades [77–84, 88, 89]. Most of the studies specifically focused on their interplay, corroborating HT role in CM evolution [77, 81, 83]. Data are supported by the large samples analyzed; nonetheless, relevant limitations, mainly related to study and concept design, do not allow to draw definite conclusions on the topic.

The ultimate existence of a causal role between HT and CM is biased by the cross-sectional design of most studies which poses several interpretational issues [31, 78–82, 87]. Cross-sectional design is not the most suitable to support a unidirectional relationship. As an example, in a cross-sectional study patient’s hypertension secondary to medication overuse, current or past, cannot be certainly excluded. Moreover, the strength of large sample populations can also conceal the lack of proper inclusion criteria with the intrinsic risk of a less accurate amnestic evaluation, especially when self-reported data are analyzed [77, 78, 80, 81, 83, 89]. In this context, the coexistence of concomitant risk factors for migraine chronicization cannot be certainly excluded.

## Conclusions

Several preclinical and clinical studies support the existence of an association between migraine and HT through multiple mechanisms, and suggest the involvement of HT in the process of transition from episodic to CM. However, the design limitations of the existing studies do not allow to draw definitive conclusions. Prospective longitudinal studies properly designed are needed to clearly define the role of HT in chronic migraine evolution and its clinical relevance as a therapeutic target in CM prevention.

## Data Availability

There are no original data.
